# Jejunal-Ileal Diverticulosis Induced Witzel Tube Failure: A Rare Cause of Small Bowel Obstruction

**DOI:** 10.7759/cureus.24209

**Published:** 2022-04-17

**Authors:** Innocent Lutaya, Ilana Logvinsky, Molly E Mefford, Nway Nway, Abid Qureshi

**Affiliations:** 1 Internal Medicine, American University of Antigua, New York City, USA; 2 Medical School, American University of Antigua, New York City, USA; 3 Obstetrics and Gynaecology, American University of Antigua, New York City, USA; 4 Internal Medicine, Interfaith Medical Center, New York City, USA; 5 Surgery, The Brooklyn Hospital Center, New York City, USA

**Keywords:** jejunal diverticular, jejunal-ileal, sbo, small bowel obstruction, witzel tube, witzel tube failure, jejunostomy, jejunostomy tube

## Abstract

Jejunal diverticulitis is a rare form of diverticulosis that occurs in the jejunum. Ileal diverticula are a type of false diverticula that are mostly asymptomatic and are usually discovered on imaging as incidental findings. Jejunal diverticula are typically difficult to diagnose pre-operatively due to their indolent and asymptomatic nature. The etiology of this condition is unclear, although some are believed to be genetic if diffuse. When symptomatic, patients may present with vague symptoms. This requires a high index of clinical suspicion because imaging results are usually negative. Management often requires surgical intervention in the presence of complications. Our case highlights a rare case of jejunal-ileal diverticulosis with inward involution causing Witzel tube (jejunostomy tube, or J-tube) obstruction and failure, along with partial obstruction of the small bowel.

## Introduction

Jejunal diverticulum consists of mucosa and submucosa (false diverticula) [1,2]. Although the etiology of this condition is unclear, intestinal dysmotility and high intraluminal pressures may be contributing factors [1]. Jejuno-ileal diverticula are extremely rare and are discovered in between 0.5%-2.3% of cases using radiographic imaging and 7% of cases on autopsy. However, it is believed that these numbers are likely to underestimate the actual frequency [3]. They are commonly asymptomatic and present with symptoms in the setting of complications such as diverticulitis, perforation, or obstruction. We present a rare instance of Witzel tube (jejunostomy or J-tube) obstruction and failure secondary to jejunal-ileal diverticulosis.

## Case presentation

An 89-year-old African American woman with a past medical history of hypertension, hyperlipidemia, and a gastric ulcer status-post removal by open subtotal gastrectomy presented to the emergency department with shortness of breath, cough, and associated halitosis. Per the nursing home, the patient had experienced dysphagia with frequent aspiration. A physical exam showed two large scars in the right upper quadrant. Further information was not obtained due to the patient being demented at baseline. A barium swallow was inconclusive. An esophagogastroduodenoscopy (EGD) was performed, which showed a large cervical esophageal diverticulum. This suggested a Zenker’s diverticulum. Further workup suggested severe aspiration pneumonia of both lungs. Her clinical presentation and imaging prompted a nutritional intervention. The best avenue for nutritional feeding at this point was jejunostomy tube placement, possibly for the rest of her life, in consideration of her age and mental status. The patient was made nothing by mouth (NPO), and general surgery was consulted. 

A decision was made to go forward with surgical intervention with an open exploratory laparotomy for insertion of a Witzel feeding Jejunostomy tube as well as lysis of adhesions. Intraoperatively, numerous large (~3cm) small-bowel diverticula extending from the ligament of Treitz to the distal jejunum were observed. In the terminal ileum, there were 4 or 5 (~1cm) small-bowel diverticula filled with hard impacted stool. Other notable findings included multiple adhesions in the stomach due to previous mesh repairs of two anterior abdominal wall hernias and subtotal gastrectomy. All the adhesions were removed with LigaSure (Medtronic, Dublin, Ireland) to prevent further complications, along with segmental resection of the proximal jejunum with primary end-to-end anastomosis due to a weak diverticular that would have ruptured soon.

**Figure 1:**
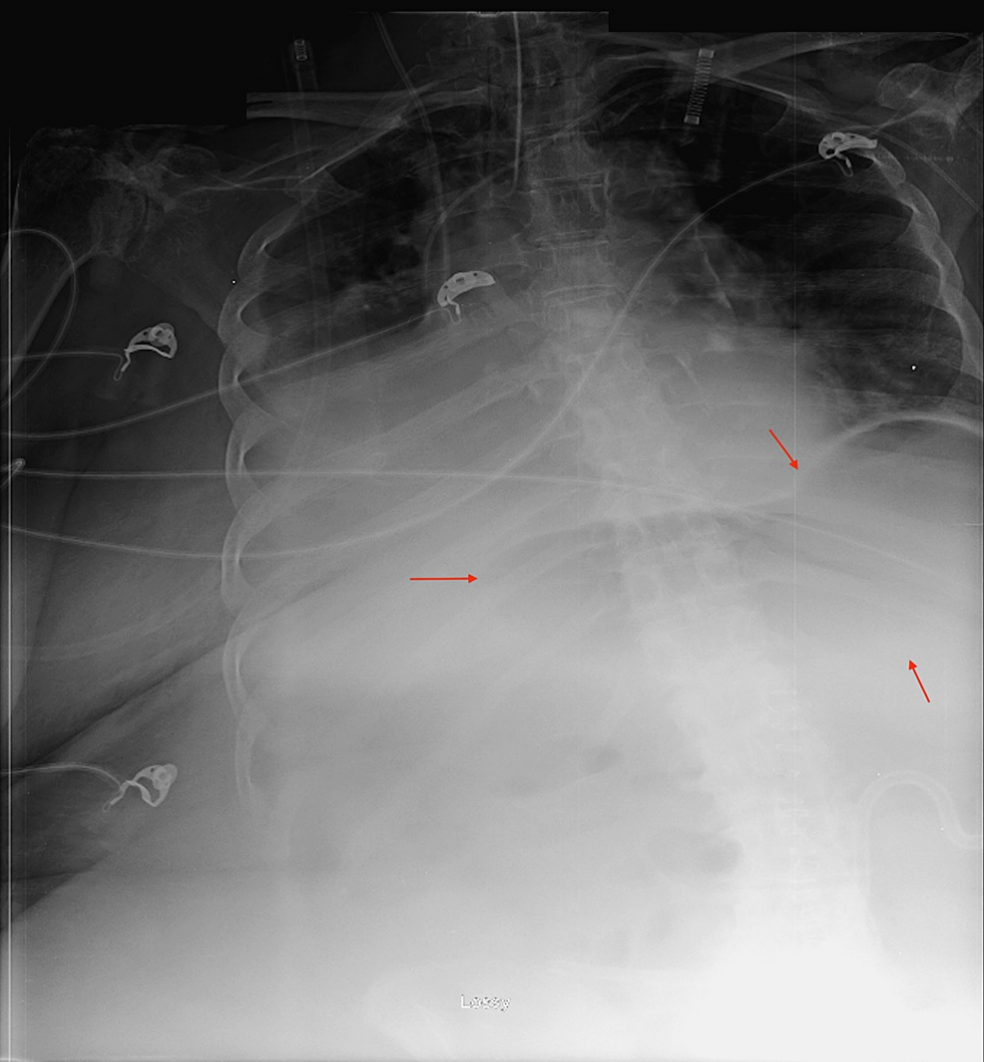
Upright abdominal radiograph demonstrating gaseous distention of the stomach with no free air identified

**Figure 2:**
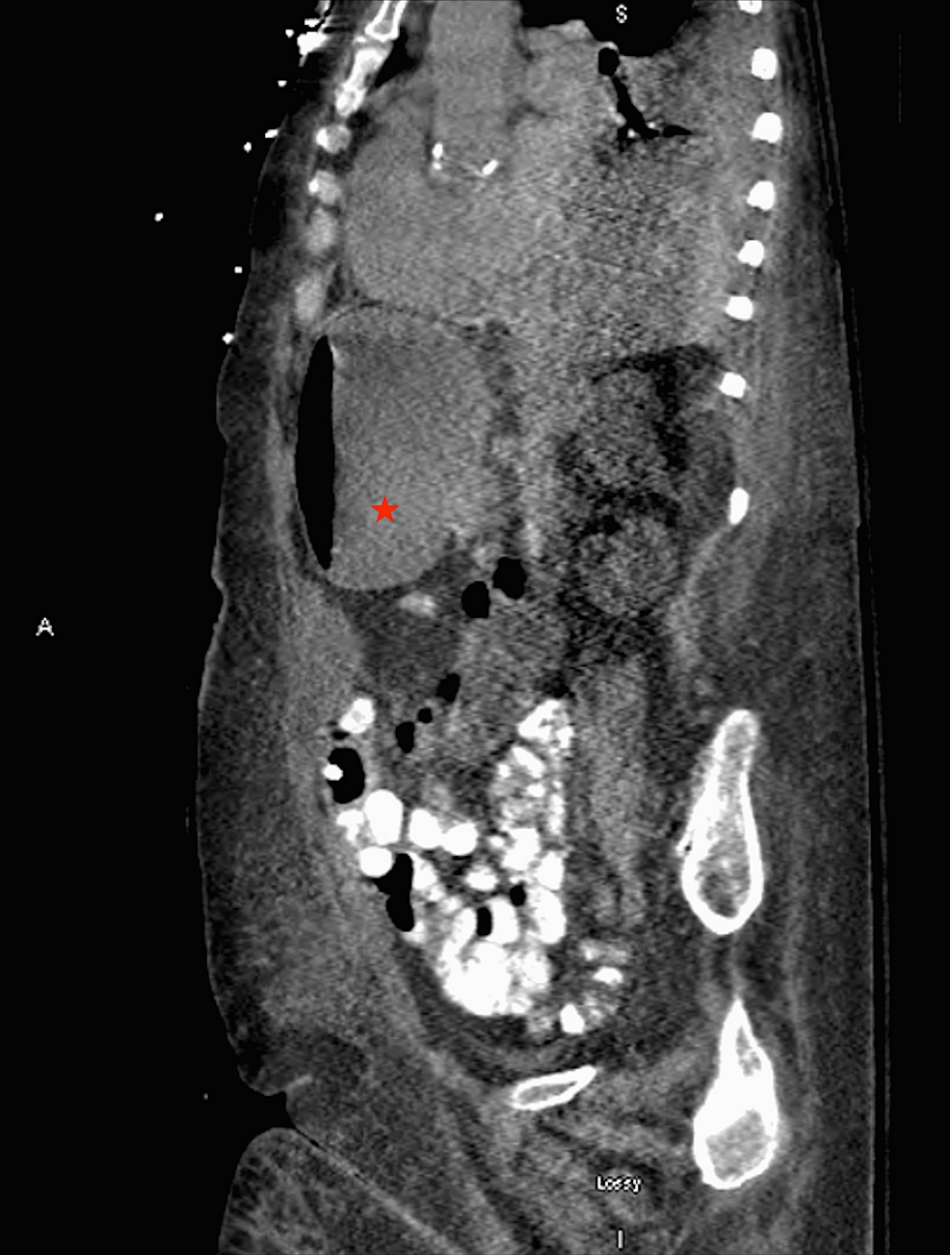
Sagittal abdominal and pelvic CT scan with enteric contrast demonstrating nonspecific gastric and duodenal distention (star) with no evidence of an obstruction given that contrast is seen within the distal bowel

Upon jejunostomy tube placement, the patient was in stable condition and transferred to the floor postoperatively. She became hypoxic overnight and was subsequently transferred to the intensive care unit (ICU) for further management of respiratory failure secondary to aspiration of gastric contents. Initially, the patient was tolerating tube feedings; however, on post-op day nine, she started exhibiting signs of small bowel obstruction with a distended abdomen, along with poor J-tube intake, which suggested a possible J-tube failure. An abdominal X-ray showed gaseous distention of the stomach with no free air in the small bowel (Figure 1). The following day, the contrast was passed through the J-tube in preparation for a follow-up CT scan to further assess bowel obstruction. There was no evidence of bowel obstruction, given that contrast was seen in the distal colon; however, a partial obstruction was not ruled out due to the patient’s clinical presentation. Other CT findings were nonspecific gastric and duodenal distention (Figure 2).

Given the high index of suspicion for small bowel obstruction, a decision was made to take the patient back to the operating room (OR) for an exploratory laparotomy with Witzel jejunostomy tube takedown, resection of the jejunum with side-to-side primary jejunal anastomosis, and placement of a Stamm gastrostomy tube. Intraoperatively it was found that one of the diverticular had involuted inward obstructing the Witzel tube, along with partial obstruction of the small bowel. This explains why our patient had signs of J-tube failure, with minimal signs of small bowel obstruction, which were supported by negative imaging findings for small bowel obstruction. Despite all that was done, the patient expired 15 days later due to complications of severe Zenker’s diverticulum-related aspiration pneumonia.

## Discussion

A diverticulum is a sac-like protrusion from the gastrointestinal bowel wall that can occur in the small and large bowel. In most cases, small diverticula are found incidentally and with no symptoms. Although rare, the symptoms can be severe and life-threatening. Jejunal diverticulum consists of mucosa and submucosa (false diverticula). Atrophic changes on the mesenteric side of the bowel may result in the formation of diverticula in patients with visceral myopathy as a result of atrophy of the jejunal wall and increased luminal pressure. The supplying blood vessels, the vasa recta, penetrate the bowel wall, causing the mucosa to protrude [1,2]. Although the etiology of this condition is unclear, intestinal dysmotility and high intraluminal pressures may be contributing factors. A variety of intestinal dysmotility conditions, such as progressive systemic sclerosis, visceral neuropathies, and myopathies, are commonly associated with them. Some cases might be inherited, as shown by the case series describing family aggregation [1]. Jejunoileal diverticula are extremely rare and are discovered between 0.5%-2.3% using radiographic imaging and 7% on autopsy. However, it is believed that these numbers are likely to underestimate the actual frequency [3].

Up to 10% of patients report complications that arise due to jejunoileal diverticula [4]. Jejunal diverticular are sometimes not seen on CT scans as was with our case; however, on abdominal CT scans, the complications of diverticulitis or small bowel obstruction can be seen. Free air might be visible in patients with peritonitis [5]. Acute diverticulitis affects approximately 4% of patients with diverticulosis [6]. 17% of individuals admitted to the hospital with acute diverticulitis develop diverticular abscesses [7]. Perforation with widespread peritonitis can occur when a diverticular abscess ruptures or when an inflammatory diverticulum ruptures freely. Perforation occurs in about 2.3%-6.4% of the cases [8]. Hematemesis and melena are common symptoms of duodenal diverticula hemorrhage, although hematochezia is more common in jejunoileal diverticula [9]. Selective mesenteric angiography or CT angiogram can be used to locate active bleeding in cases of jejunal diverticular hemorrhage [10]. 

In the presence of acute complications of the jejunal diverticula, such as diverticulitis, major bleeding, or bowel perforation, the treatment of choice is resection (laparoscopic or open) of the affected segment of the small bowel with primary end-to-end anastomosis. An exception is a pan-jejunoileal diverticulosis, for which conservative treatment may be preferred. This is because surgery could lead to severe malnutrition and possibly death. A jejunal diverticulum is difficult to diagnose because of its low incidence, asymptomatic indolent course, poor clinical index of suspicion, and imprecise diagnostic imaging in emergency conditions. This has enormous ramifications for prompt and effective treatment and can result in significantly higher morbidity and mortality with the onset of complications [11]. Treatment is not required for those who are asymptomatic. Conservative measures are recommended in symptomatic patients with complaints of chronic vague abdominal symptoms, diarrhea, and malabsorption unless large diverticula are found [12]. Untreated diverticulitis can lead to repeated gastrointestinal bleeding and perhaps death [8,12-15,16]. 

Decompression is usually initiated for small bowel obstruction. However, the modality of decompression or nutrition may lead to bowel obstruction even among patients that lack prior evidence of obstruction. Additionally, the jejunal diverticula may create weakness within the intestinal wall, which could be prone to rupture. Due to the minimal research and literature studies regarding Witzel tube obstruction, the presented case shows a rare complication of Witzel tube failure that can cause small bowel obstruction. In our case, the jejunal-ileal diverticula led to J-tube obstruction. For these reasons, we believe that surgeons should be cautious when choosing to use Witzel, nasojejunal, or any tube that extends beyond the duodenum. 

## Conclusions

In summary, jejunal diverticula are usually asymptomatic and are an incidental finding that’s usually hard to image. When symptomatic, patients can present with vague acute symptoms necessitating surgical intervention. This requires a high degree of clinical suspicion even in the face of negative imaging results like our patient. Complications include obstruction, diverticulitis, and perforation. Our case highlights a rare case of diverticular involuting, inward causing Witzel tube obstruction and failure, along with partial obstruction of the small bowel. 

## References

[1] Koch AD, Schoon EJ (2007). Extensive jejunal diverticulosis in a family, a matter of inheritance?. Neth J Med.

[2] Krishnamurthy S, Kelly MM, Rohrmann CA, Schuffler MD (1983). Jejunal diverticulosis: a heterogenous disorder caused by a variety of abnormalities of smooth muscle or myenteric plexus. Gastroenterology.

[3] De Peuter B, Box I, Vanheste R, Dymarkowski S (2009). Small-bowel diverticulosis:imaging findings and review of three cases. Gastroenterol Res Pract.

[4] Eckhauser FE, Zelenock GB, Freier DT (1979). Acute complications of jejuno-ileal pseudodiverticulosis: surgical implications and management. Am J Surg.

[5] Snyder MJ (2004). Imaging of colonic diverticular disease. Clin Colon Rectal Surg.

[6] Shahedi K, Fuller G, Bolus R (2013). Long-term risk of acute diverticulitis among patients with incidental diverticulosis found during colonoscopy. Clin Gastroenterol Hepatol.

[7] Ambrosetti P, Chautems R, Soravia C, Peiris-Waser N, Terrier F (2005). Long-term outcome of mesocolic and pelvic diverticular abscesses of the left colon: a prospective study of 73 cases. Dis Colon Rectum.

[8] de Bree E, Grammatikakis J, Christodoulakis M, Tsiftsis D (1998). The clinical significance of acquired jejunoileal diverticula. Am J Gastroenterol.

[9] Johnson KN, Fankhauser GT, Chapital AB, Merritt MV, Johnson DJ (2014). Emergency management of complicated jejunal diverticulosis. Am Surg.

[10] Yaqub S, Evensen BV, Kjellevold K (2011). Massive rectal bleeding from acquired jejunal diverticula. World J Emerg Surg.

[11] Nejmeddine A, Bassem A, Mohamed H, Hazem BA, Ramez B, Issam BM (2009). Complicated jejunal diverticulosis: a case report with literature review. N Am J Med Sci.

[12] Tsiotos GG, Farnell MB, Ilstrup DM (1994). Nonmeckelian jejunal or ileal diverticulosis: an analysis of 112 cases. Surgery.

[13] Butler JS, Collins CG, McEntee GP (2010). Perforated jejunal diverticula: a case report. J Med Case Rep.

[14] Kassir R, Boueil-Bourlier A, Baccot S (2015). Jejuno-ileal diverticulitis: etiopathogenicity, diagnosis and management. Int J Surg Case Rep.

[15] Syllaios A, Koutras A, Zotos PA (2018). Jejunal diverticulitis mimicking small bowel perforation: case report and review of the literature. Chirurgia (Bucur).

[16] Longo WE, Vernava AM 3rd (1992). Clinical implications of jejunoileal diverticular disease. Dis Colon Rectum.

